# “Because if we talk about health issues first, it is easier to talk about human trafficking”; findings from a mixed methods study on health needs and service provision among migrant and trafficked fishermen in the Mekong

**DOI:** 10.1186/s12992-018-0361-x

**Published:** 2018-05-09

**Authors:** Nicola S. Pocock, Reena Tadee, Kanokwan Tharawan, Wansiri Rongrongmuang, Brett Dickson, Soksreymom Suos, Ligia Kiss, Cathy Zimmerman

**Affiliations:** 1 United Nations University International Institute of Global Health, UKM Medical Centre, Jalan Yaacob Latif, Bandar Tun Razak, 56000 Cheras, Kuala Lumpur, Malaysia; 2 Institute for Population and Social Research, Mahidol University, Salaya, Phutthamonthon District, Nakhon Pathom, 73170 Thailand; 3 Independent consultant, Bangkok, Thailand; 4 International Organization for Migration, Norodom Blvd, No. 281, 4th Floor, Sangkat Tonle Basac, Khan Chamkamorn, Phnom Penh, Cambodia; 5 Independent consultant, Phnom Penh, Cambodia; 6 Department of Global Health & Development, Faculty of Public Health and Policy, London School of Hygiene and Tropical Medicine, 15-17 Tavistock Place, Kings Cross, London, WC1H 9SH UK

**Keywords:** Human trafficking, Forced labour, Thailand, Cambodia, Myanmar, Trafficked fishermen, Migrant fishermen, Fishing, Migrant health

## Abstract

**Background:**

Human trafficking in the fishing industry or “sea slavery” in the Greater Mekong Subregion is reported to involve some of the most extreme forms of exploitation and abuse. A largely unregulated sector, commercial fishing boats operate in international waters far from shore and outside of national jurisdiction, where workers are commonly subjected to life-threatening risks. Yet, research on the health needs of trafficked fishermen is sparse. This paper describes abuses, occupational hazards, physical and mental health and post-trafficking well-being among a systematic consecutive sample of 275 trafficked fishermen using post-trafficking services in Thailand and Cambodia. These findings are complemented by qualitative interview data collected with 20 key informants working with fishermen or on issues related to their welfare in Thailand.

**Results:**

Men and boys trafficked for fishing (aged 12–55) were mainly from Cambodia (*n* = 217) and Myanmar (*n* = 55). Common physical health problems included dizzy spells (30.2%), exhaustion (29.5%), headaches (28.4%) and memory problems (24.0%). Nearly one-third (29.1%) reported pain in three or more areas of their body and one-quarter (26.9%) reported being in “poor” health. Physical health symptoms were strongly associated with: severe violence; injuries; engagement in long-haul fishing; immigration detention or symptoms of mental health disorders. Survivors were exposed to multiple work hazards and were perceived as disposable when disabled by illness or injuries. Employers struggled to apply internationally recommended Personal Protective Equipment (PPE) practices in Thailand. Non-governmental organizations (NGOs) encountered challenges when trying to obtain healthcare for uninsured fishermen. Challenges included fee payment, service provision in native languages and officials siding with employers in disputes over treatment costs and accident compensation. Survivors’ post-trafficking concerns included: money problems (75.9%); guilt and shame (33.5%); physical health (33.5%) and mental health (15.3%).

**Conclusion:**

Fishermen in this region are exposed to very serious risks to their health and safety, and their illnesses and injuries often go untreated. Men who enter the fishing industry in Thailand, especially migrant workers, require safe working conditions and targeted protections from human trafficking. Survivors of the crime of sea slavery must be provided with the compensation they deserve and the care they need, especially psychological support.

**Electronic supplementary material:**

The online version of this article (10.1186/s12992-018-0361-x) contains supplementary material, which is available to authorized users.

## Background

The trafficking of fishermen has emerged as a growing phenomenon in Asia-Pacific [1–5]. Beyond the myriad of occupational risks, including drowning, injuries from operating heavy equipment, fatigue from long working hours, and sleep deprivation from poor weather conditions [6, 7], men trafficked for commercial fishing are especially vulnerable to exploitation and abuse because of the isolation in deep off-shore, mobile worksites which evade authorities’ interventions. Men trafficked for long-haul fishing may be stranded on vessels for months or years, made possible by transshipment, whereby cargo vessels re-supply commercial fishing boats and pick up their catches, obviating the need to dock in port and making escape almost impossible for trafficked men on board [5, 8, 9]. There have been reports of men who, in desperation, jump overboard and drown while fleeing vessels [4]. Even when boats eventually dock at overseas ports, international security regulations often require foreign crew to stay aboard vessels, preventing access to medical care and stopping their escape [10].

Trafficked fishermen report working 18–24 h days with limited to no rest, poor occupational safety and health (OSH) and violence [1, 8, 11, 12]. Among a sample of fishermen who were defined as forced labour or trafficked, reports of physical abuse ranged from 10%–50%, [11–13] with some of the most extreme violence documented among long-haul fishermen [8, 13, 14]. Men are threatened and beaten with weapons or may be cast overboard by superiors when sick or non-compliant. Men who were regularly physically beaten have contemplated or acted on suicidal thoughts [8, 11, 12, 14].

This study examines health needs and service provision for trafficked and migrant fishermen. In this paper, we use the term “trafficked fishermen” if males have been formally identified as trafficked by authorities. We use the term “migrant fishermen” to describe migrant males who are working in Thailand in the fishing sector, who are not formally identified as trafficked by authorities, some of whom may have experienced some elements of exploitation or trafficking (but not always).

### Physical and mental health among labour-trafficked men and associated factors

Health problems among labour-trafficked men have included headaches, back pain, fatigue, depression, anxiety and PTSD [15]. In addition to violence and restricted freedom during trafficking, mental health disorders among labour-trafficked men in the UK were associated with post-trafficking factors, namely fear of traffickers, absence of a confidante and pre-trafficking sexual violence [16].

Although most literature focuses on the abuses that occur while individuals are in the trafficking situation, previous work has identified multiple health risks that are associated with the post-trafficking period, including during immigration detention and re-trafficking following escape [17]. Trafficked fishermen who manage to escape are frequently detained in immigration detention centres. Many are not formally identified as victims of trafficking and some have been re-trafficked [1, 8, 12, 18]. When detained by authorities and not identified as trafficking victims, men typically must pay fees or bribes to speed up the deportation process or cover their travel expenses home, which is an additional source of stress [1]. Findings from research with asylum seekers and refugees on the health impacts of immigration detention may be relevant for trafficked persons who have experienced trauma. Evidence suggests that among asylum seekers who were torture survivors, immigration detention is linked to mental health disorders, suicidal ideation and self-harm, as well poor physical health from unsanitary conditions and assaults from immigration officers [19].

Somatic symptoms of poor physical health among migrants or trafficked persons may indicate psychological distress, or may result from prolonged exposure to poor conditions, violence and untreated injury [20, 21]. Post-traumatic responses reported by Cambodian migrant workers included: headaches; sleeplessness; dizziness and appetite loss, which were indicated by cultural idioms of distress including “sadness”, “thinking too much” and “worry in the heart” [22]; somatic symptoms were also common among traumatized Cambodian refugees [23, 24]. Symptoms of poor physical or mental health can worsen without access to care.

### Healthcare seeking and services for migrant fishermen in Thailand

Both undocumented migrants and those with work permits may avoid seeking care for fear of arrest or deportation, or they may internalize exclusionary arguments that they are “undeserving” [25, 26], which usually leads to high rates of self-treatment or use of private clinics [27, 28]. Among migrant fishermen in Thailand, higher proportions of Burmese and Khmer fishermen self-treated compared to Thai fishermen [29].

Migrant fishermen in Thailand can enrol in the Health Insurance Card Scheme (HICS), administered the Ministry of Public Health (MOPH). This benefits package includes inpatient and outpatient care, and notably ARV treatment [30]. In 2014, the premium was 2100 THB (66 USD) and 600THB (19 USD) for a pre-employment health screening [31], paid annually by either employer or employee, although the coverage period has now been extended to two years at a cost of 3200 THB (100 USD) and 500 THB (16 USD) respectively [32].

Undocumented migrants can enrol in the HICS, although some hospitals have requested that would-be enrolees show official documents including residence cards [33]. A health screening is conducted alongside registration for temporary work permits (pink card), which restricts migrant workers’ movement to their area of employment [26]. Migrants with the pink card are then eligible to apply for temporary passports as part of the Nationality Verification (NV) process, where they then apply for a new work permit with permission to remain legally for two years (renewable up to a maximum of four years) [34]. Blue work permits were issued to migrant workers in land-based sectors, while orange work permits were issued to migrant workers in fishing [35]. At the time of data collection, fishermen were unable to switch their employment to land-based sectors, ostensibly to discourage them from leaving the fishing sector which suffers from labour shortages [35, 36].

When this study was conducted in 2014, the HICS was restricted to the hospital where the card was obtained and was not portable between provinces or hospitals, which was problematic for fishermen docking at different provincial ports. Not all migrants enrol, or some enrol but subsequently drop out of the scheme [34]. HICS has improved migrants’ access to services and reduced out-of-pocket payments (OPPs), but outpatient utilization rates have remained low. Migrants primarily used only inpatient services, which meant that there were high self-treatment rates and many delayed seeking care [30, 37, 38]. The Social Security Scheme (SSS) provides similar levels of health coverage as the HICS for migrants who entered Thailand via a bilateral Memorandum Of Understanding (MOU) with neighbouring countries, or whom have had passports issued following Nationality Verification. However, under this scheme, both employers and employees must make monthly contributions, and migrants cannot avail of retirement or unemployment benefits despite paying for these. Furthermore, fishermen are effectively ineligible as “informal” sectors including fishing are excluded from the SSS [34, 39]. Provision of health and support services differs for persons formally identified as trafficked by authorities.

### Post-trafficking services for trafficked fishermen in Thailand and Cambodia

Trafficked fishermen formally identified by Thai authorities are sent to one of four shelters for male trafficking survivors, where food, accommodation and vocational training are provided alongside medical care, assistance with legal cases, repatriation and reintegration support [40]. Cambodian trafficked fishermen identified overseas are mainly repatriated via IOM’s Assisted Voluntary Return and Reintegration (AVRR) scheme, to temporary accommodation in Phnom Penh where they receive a medical check-up including psychological evaluation, clothing, a cash grant and travel support to return home [41]. Most, if not all, Cambodian men return to deprived rural areas, sometimes with serious psychological conditions, with few or no health services or where they require payments which they often cannot afford. Many suffer with untreated conditions [18]. This study presents the first health data on trafficked fishermen from a large sample and describes how health and welfare providers reach migrant and potentially trafficked fishermen.

## Methods

Analyses were conducted in an exploratory, sequential design. Quantitative data on occupational risks and health needs were analysed first. Subsequent qualitative data analysis explored themes that would benefit from a more in-depth understanding of key topics that were absent from the quantitative data, such as strategies used by providers to reach fishermen and challenges in providing care. While analyses were conducted separately, quantitative and qualitative findings are reported together in Results by theme.

### Quantitative data collection

Quantitative survey data were analysed from structured interviews with 275 male survivors of trafficking for commercial fishing who were in the care of post-trafficking services (as part of a larger study) [42]. The questionnaire was adapted from an instrument used in a prior study with trafficked women using post-trafficking services in Europe [43], to include a wider range of labour sectors. The questionnaire was translated into Khmer, Thai, Vietnamese, Burmese, and Lao, refined through group discussions with IOM counter-trafficking teams, further revised through pilot-testing, and reviewed after back-translation into English [42].

The sample was selected in two stages; first, 15 post-trafficking services were selected based on relationship to IOM country teams and agreements with government agencies. Second, a consecutive sample of service users were asked to participate in interviews carried out by shelter staff within 2 weeks of service admission between December 2011 and May 2013. Trafficked fishermen (including boys) were using either a post-trafficking service in Cambodia or Thailand. Written informed consent was obtained from participants prior to interview; for those aged below 18, the child’s care team were also consulted. A strict ethics protocol based on the World Health Organization Ethical Recommendations for Interviewing Trafficked Women was followed [44]. All interviewers had the professional training or experience to identify when an individual should not be interviewed, when to stop or pause an interview, how to respond to distress and when to make necessary referrals. During consent procedures, the interviewer explained the study content and option to refuse or interrupt participation without consequences for services provision [45, 46]. Ethics approval was obtained from the Ministry of Social Development and Human Security in Thailand, the National Ethics Committee for Health Research in Cambodia and the London School of Hygiene and Tropical Medicine (LSHTM).

### Data coding and analysis

Physical health was assessed using an adapted version of the Miller Abuse Physical Symptom and Injury Scale for abuse-specific health problems [47]. A binary variable was created based on a participant endorsing “quite a lot” or “extremely” for each physical health symptom. Binary variables were created for experiencing poor self-assessed health and experiencing 3 or more areas of pain. The 4 most commonly reported physical health symptoms, poor self-assessed health and reporting 3 or more areas of pain were selected as response variables in bivariable analysis.

The occupational health risks (OHR) score was created by combining binary variables on occupational hazard exposures and presence of personal protective equipment for that hazard [46]. Fishermen were categorized as “long-haul” if they were trafficked to Indonesia, Malaysia, Mauritius or South Africa, and “short-haul” if they were trafficked to Thailand, following consultation with study partners. Binary variables were created for ever experiencing a serious injury and ever being detained by authorities. Questions on abuse were derived from the violence and health outcome modules of the WHO multi-country study on intimate partner violence [48]. Violence was classified as less severe if it involved slaps, pushes and hits, and more severe if it included: being kicked, dragged or beaten up; tied or chained; choked or burned; released a dog to bite or scratch; being threatened with a weapon; cut with a knife, shot or forced to have sex [49, 50]. The living situation score was the sum of 9 endorsed items; the post-trafficking concern score summed endorsed items from 12 concerns. Mental health was assessed using the Hopkins Symptoms Checklist 25 for depression and anxiety and the Harvard Trauma Questionnaire for post-traumatic stress (PTSD) [51–53]. Binary variables for being symptomatic of anxiety, depression and PTSD were calculated based on cut-off scores of 1.75, 1.625 and 2.0 respectively, as described elsewhere [42].

Unadjusted odds ratios were calculated using logistic regression to explore the relationship between theorized exposures that may impact physical health (see Conceptual Framework A in Additional file 1). We conducted bivariable rather than multivariable analysis due to small sample sizes for the health outcomes and because earlier construction of a Directed Acyclic Graph indicated that our multivariable analysis would be biased due to the direction of the relationships between some theorized predictors [54, 55]. Continuous variables for hours worked/day, OHR score, living situation score and post-trafficking concerns score were rescaled on the interquartile range to aid interpretation in bivariable analyses, where the odds ratio allows comparison between a person with a typical “high” value on the predictor to a person with a typical “low” value [56]. Analysis was conducted in Stata 14.

### Qualitative data collection

A provincial port research site and major fishing hub was chosen as the primary location to interview health and welfare providers and fishery associations, followed by Bangkok, where international organizations were usually based. The port research site is not named to preserve the anonymity of participants.

The topic guide was developed based on themes from a conceptual framework conceived for the study (see Conceptual Framework B in Additional file 1). Semi-structured interviews were conducted with key informants between August to October 2014 working either directly with fishermen or on issues related to their welfare (Table 1). Purposive and snowball sampling were used to recruit participants, based on an initial sample frame of service providers compiled from reviewing reports/policy documents. Written informed consent was obtained from each participant. Most interviews were conducted in Thai with a research assistant interpreter, who was trained in interview techniques and topic guide content. Interviews were digitally recorded, transcribed verbatim to English or Thai and subsequently translated to English. Ethics approval was obtained from the Institute of Population and Social Research, Mahidol University, Thailand and the LSHTM.

**Table 1:** Participants interviewed for qualitative sample (*n* = 20)

Organization type	Number
NGO health and welfare providers (NGO)	10
Government health and welfare providers (HSP)	4
Fishery associations (FA)	3
International organizations (IO)	3
Total	20

### Qualitative data coding and analysis

Qualitative data were analysed using thematic analysis. Transcripts were read and re-read to familiarize with the data and generate initial codes. A priori themes identified from the topic guides and conceptual framework (deductive approach) shaped earlier versions of a coding framework. New codes were also identified from the data (inductive approach). Taken together, emergent codes and a priori codes were collapsed and collated under overarching themes. Themes were continually reviewed for internal consistency and distinguishability from other themes until refined themes were developed, taking care to include negative cases and less prominent themes [57]. Qualitative data were coded and analysed by hand initially, before being coded and sorted in NVivo 11. Finally, memos were developed by theme in OneNote.

## Results

### Participant characteristics of trafficked fishermen

Most long-haul fishermen were from Cambodia (99.0%) accessing services in Cambodia, while most short-haul fishermen were from Myanmar (71.4%) accessing services in Thailand (Table 2). Most fishermen (44.7%) were aged 25 to 34. Long-haul fishermen were mainly trafficked to Indonesia (65.2%) and spent much longer time in trafficking situations compared to fishermen on short-haul boats (median 23 and 5 months respectively). Many long-haul fishermen (43.9%) reported that they had been detained by authorities compared to 27.3% of short-haul fishermen. Long-haul fishermen spent a median of one month in immigration or police detention compared to 10 days among short-haul fishermen (Table 2).

**Table 2:** Participant characteristics, fishermen using post-trafficking services in Cambodia and Thailand (*n* = 275)

	Long-haul fishermen (*n* = 198)		Short-haul fishermen (*n* = 77)		Whole sample (*n* = 275)	
	Number	Percent	Number	Percent	Number	Percent
Age						
10 to 14	–	–	1	1.3%	1	0.4%
15 to 17	6	3.0%	6	7.8%	12	4.4%
18 to 24	71	35.9%	32	41.6%	103	37.5%
25 to 34	96	48.5%	27	35.1%	123	44.7%
> = 35	25	12.6%	11	14.3%	36	13.1%
Education						
Primary or less (1–5 grade)	97	49.0%	39	50.6%	136	49.5%
Secondary (6–8 grade)	42	21.2%	21	27.3%	63	22.9%
Higher (10–11 grade)	4	2.0%	6	7.8%	10	3.6%
University degree	–	–	3	3.9%	3	1.1%
No formal education	55	27.8%	8	10.4%	63	22.9%
Country of destination						
China	2	1.0%	–	–	2	0.7%
Malaysia	28	14.1%	–	–	28	10.2%
Thailand	0	0.0%	77	100.0%	77	28.0%
Indonesia	129	65.2%	–	–	129	46.9%
Mauritius	33	16.7%	–	–	33	12.0%
South Africa	6	3.0%	–	–	6	2.2%
Home country						
Cambodia	196	99.0%	21	27.3%	217	78.9%
Myanmar	–	–	55	71.4%	55	20.0%
Thailand	2	1.0%	–	–	2	0.7%
Can’t remember	–	–	1	1.3%	1	0.4%
Time in trafficking (months)						
< 1	1	0.5%	7	9.1%	8	2.9%
1 to 6	25	12.6%	33	42.9%	58	21.1%
7 to 12	22	11.1%	14	18.2%	36	13.1%
13 to 23	50	25.3%	12	15.6%	62	22.5%
> = 24	99	50.0%	8	10.4%	107	38.9%
Missing data	1	0.5%	3	3.9%	4	1.5%
Median months in trafficking (median absolute deviation)	197	23.0 (13.0)	74	5.1 (3.8)	271	16.0 (11.5)
Speaks language of destination country	198	49.0%	77	20.8%	275	41.1%
Previous experience in sector	15	7.6%	3	3.9%	18	6.6%
Country of service access						
Cambodia	198	100.0%	21	27.3%	219	79.6%
Thailand	–	–	56	72.7%	56	20.4%
Ever detained by authorities in destination country	87	43.9%	21	27.3%	108	39.3%
Time in detention (months)						
<=1	39	44.8%	14	66.7%	53	49.1%
2 to 5	35	40.2%	6	28.6%	41	38.0%
> = 6	13	14.9%	1	4.8%	14	13.0%
Median months in detention (median average deviation)	87	1.0 (0.9)	21	0.3 (0.3)	108	1.0 (0.9)

### Occupational hazards, safety, violence and abuses at sea

Half (49.5%) of trafficked long-haul fishermen incurred at least one serious injury compared to 40.0% of short-haul fishermen (Table 3). Common accidents and injuries described by NGO participants assisting men included severed limbs, injuries from rope pulleys, winches and sharp fish bones. Other hazards included inhaling poisonous fumes from the fish storage room and men falling off the boat accidentally:

**Table 3:** Occupational hazards, abuses and healthcare during trafficking among fishermen using post-trafficking services in Cambodia and Thailand (n = 275)

	Long-haul fishermen (*n* = 198)		Short-haul fishermen (*n* = 77)		Whole sample (*n* = 275)	
	Number	Percent	Number	Percent	Number	Percent
Occupational hazards (selected)						
Unstable or heavy work platforms	169	85.4%	57	74.0%	226	82.2%
Work along rocky coasts or in remote offshore	111	56.1%	68	88.3%	179	65.1%
Small, unstable or badly maintained fishing vessel	56	28.3%	38	49.4%	94	34.2%
Badly maintained or no fishing equipment	48	24.2%	29	37.7%	77	28.0%
No safety/bad or no survival equipment	105	53.0%	65	84.4%	170	61.8%
Long hours in the sun, cold or wet without a break	189	95.5%	77	100.0%	266	96.7%
Protective gear						
Sun hat	76	38.4%	63	81.8%	139	50.6%
Gloves	151	76.3%	24	31.2%	175	63.6%
Life vest	59	29.8%	15	19.5%	74	26.9%
No protective gear given	28	14.1%	9	11.7%	37	13.5%
Hours worked per day						
<=8	13	6.6%	2	2.6%	15	5.5%
8 to 10	0	0.0%	5	6.5%	5	1.8%
11 to 15	12	6.1%	13	16.9%	25	9.1%
16 to 19	9	4.5%	12	15.6%	21	7.6%
> = 20	103	52.0%	12	15.6%	115	41.8%
No fixed hours	61	30.8%	33	42.9%	94	34.2%
Median hours worked/day (median average deviation)^a^	137	22 (2)	44	18 (3)	181	21 (3)
Occupational health risk score (median)^b^	198	50	77	60	275	50
Worked every day^f^	192	97.5%	75	97.4%	267	97.5%
No or very few rest breaks	176	88.9%	69	89.6%	245	89.1%
No time off for sickness or holiday	172	86.9%	67	87.0%	239	86.9%
Experienced at least 1 serious injury^g^	98	49.5%	30	40.0%	128	46.9%
Injuries still cause pain/difficulty	57/98	58.2%	9/30	30.0%	66/128	51.6%
Ever needed healthcare or was injured	129	65.2%	49	63.6%	178	64.7%
Who provided medical care^d^						
Doctor	10	7.8%	2	4.1%	12	6.7%
Nurse	2	1.6%	–	–	2	1.1%
Owner/manager	44	34.1%	13	26.5%	57	32.0%
Co-worker	8	6.2%	5	10.2%	13	7.3%
Received regular health checks from trafficker/employer	6	4.7%	2	4.1%	8	4.5%
Other	2	1.6%	2	4.1%	4	2.3%
Did not receive healthcare	61	47.3%	32	65.3%	93	52.3%
Cheated of wages	140	70.7%	55	71.4%	195	70.9%
Median payment in USD/day (median average deviation)	58	$1.33 ($1.00)	21	$2.52 ($1.73)	79	$1.44 ($1.14)
Restricted freedom^e^	162	81.8%	70	90.9%	232	84.4%
No documents	151	76.3%	57	75.0%	208	75.9%
Violence severity						
No violence	61	30.8%	18	23.4%	79	28.7%
Experienced less severe violence	36	18.2%	12	15.6%	48	17.5%
Experienced more severe violence	101	51.0%	47	61.0%	148	53.8%
Living conditions						
Living and sleeping in overcrowded rooms	176	88.9%	67	87.0%	243	88.4%
Sleeping in dangerous conditions (close to generator or engine)	80	40.4%	28	36.4%	108	39.3%
Nowhere to sleep/sleeping on the floor	141	71.2%	71	92.2%	212	77.1%
Poor basic hygiene	127	64.1%	52	67.5%	179	65.1%
Inadequate water for drinking	101	51.0%	44	57.1%	145	52.7%
Insufficient food	94	47.5%	27	35.1%	121	44.0%
No clean clothing items	155	78.3%	64	83.1%	219	79.6%
Overexposure to sunlight or rain	191	96.5%	76	98.7%	267	97.1%
Other hazards	30	15.2%	21	27.3%	51	18.6%
Living situation score (mean, SD)^c^	198	5.5 (1.8)	77	5.8 (1.9)	275	5.6 (1.8)
Alcohol						
Never drank alcohol	86	43.4%	44	57.1%	130	47.3%
Drank afew times per year	82	41.1%	13	16.9%	95	34.6%
Drank afew times per month	23	11.6%	19	24.7%	42	15.3%
Drank afew times per week	4	2.0%	1	1.3%	5	1.8%
Drank everyday	3	1.5%	–	–	3	1.1%
Forced to take drugs by employer or trafficker	15	7.6%	1	1.3%	16	5.8%

^a^Among those who specified hours worked

^b^Score min = 0, max = 100

^c^Score min = 0, max = 9

^d^Among those ever injured or specifying that they needed care during trafficking

^e^Either “Never” being free or being locked in a room

^f^1 missing

^g^2 missing

“They work during the night and there are no toilets on the boat. They must walk along the keel and do their business hanging from the boat. If it is a new worker without proper skill there could be a chance that he would fall into the sea and simply disappear.” (NGO, 6).

NGOs and industry participants cited crew inexperience leading to accidents and injuries. Swimming under boats to retrieve tangled fishing nets and cutting them from the propeller was another occupational hazard, bringing risk of death among fishermen who did not know how to swim. Children were deemed particularly suited for this task by some employers because captains perceive that they were smaller and more agile to swim under the boat:

“I ask why you (captain) need children in the vessel? They say “sometimes we throw the rope, or for swimming under the fishing vessel, the children are really nice”… (they) swim, pull the rope [free].” (NGO, 13).

Most trafficked short-haul fishermen (84.4%) reported having no safety or survival equipment compared to 53.0% of long-haul fishermen (Table 3). Just 26.9% of trafficked fishermen had a life vest. Most short-haul fishermen (81.8%) had sun hats compared to 38.4% of long-haul fishermen, a higher proportion of whom received gloves (76.3%) compared to short-haul fishermen (31.2%). An industry participant spoke about the difficulties and questioned the suitability of applying global safety standards and personal protective equipment (PPE) recommendations in the Thai context:

“The ILO had meetings with us about [boat] safety. They told us that, in foreign countries, when we pull the rope, we must put on gloves and shoes. But the foreign fishery and Thai fishery are different. Sometimes wearing gloves can be dangerous as the ropes we use are fluffy and the gloves get stuck. If we don’t pull out our hands in time, it can be really dangerous… Sometimes we can’t apply some requirements with the way we work.” (FA, 21).

He went on to describe other examples of how applying Western safety standards was not suitable:

“Sometimes the ship is slippery and in Europe, the workers put on mechanic jumpsuits. But we can’t do this as the weather is really hot and it can be uncomfortable… We only put on working shoes when we go to the cold storage [room] or when we catch live fish and need to protect ourselves from them. Besides that, we don’t wear them as they are slippery and uncomfortable.” (FA, 21).

This participant alluded to PPE not only being uncomfortable in the climate, but as causing more danger. International organization (IO) participants suggested that OSH was not prioritized by employers; they did not see it as enough of a problem, and were reluctant to invest in safety measures following already large upfront costs of the boat, particularly among employers using trafficked labour:

“Safety of the workers is not their priority.” (IO, 3).

To improve outcomes, one participant said that boats would have to be redesigned completely, e.g. winches covered, requiring more investment. Other hazards included extreme working hours (median 21 h/day) among trafficked fishermen; 89.1% had no or few rest breaks. Half (52.7%) of trafficked fishermen had inadequate drinking water; 44.0% had insufficient food (Table 3). Among key informants, descriptions of food provision varied between inadequate/not fresh to unlimited fresh fish supplied. One government Health Service Provider (HSP) described the case of a fisherman returning from Indonesia with vitamin deficiencies because of a lack of vegetables, as has been observed in several cases of beriberi at Thai ports recently [58, 59]. Half (53.8%) of trafficked fishermen experienced severe violence (Table 3). Being killed and thrown overboard was sometimes threatened by superiors. Among trafficked men who were sold from boat to boat, one participant noted the toll the work would take on men and how quickly they’d fall sick:

“When they sold [a fisherman], first it’s 15,000 baht (473 USD), and work 1 year without wages… after that they sold to the second vessel, 8000 baht (252 USD). [They] work maybe 6 to 8 months, until they’re sick. No need to treatment. And they throw to the sea.” (NGO, 13).

Men were ultimately perceived as disposable once their labour and health had been exhausted. Being forced to take drugs was another abuse, experienced by 7.6% of long-haul and 1.3% of short-haul trafficked fishermen (Table 3).

### Health as an inroad to assisting trafficked men

Health and welfare NGOs’ mandates involved addressing health (primarily HIV) among migrants, not trafficking. However, their health mandate did grant NGOs access to potentially trafficked fishermen, as health was a less contentious topic for employers. When NGOs provided free medicines and health education, it allowed NGOs to become useful to employers and ultimately gain their trust:

“When we work on AIDs or health issues they are already a soft topic. We sometimes approach [employers] individually and introduce them to our drop in [centre]… The workers can come in and get treated without going to the doctor so [employers] see the benefit of the place… They want their workers to work with them for a long time without sickness or health issue or at least get treated when they do.” (NGO, 6).

This participant went on to explain how the NGO’s position of putting health before legal concerns about undocumented workers had won employers over:

“Our selling point is that they do not feel that we are harmful to them. Their concern is that they have employed illegal workers, but we assure them we understand that there are many requirements to get the workers registered... At first we were not trusted, but after a time they saw our work and started trusting us.” (NGO, 6).

Similarly, other NGO participants discussed using the “healthy employee” frame to encourage employers to invest in migrant fishermen’s health. Health provided a less controversial entry point before discussions or awareness raising with employers about trafficking could take place:

“Because if we talk about health first it is easier to talk about human trafficking. That is a serious issue. But when we mix everything in all together I think there is a better chance, I think this is a good strategy.” (NGO, 6).

Trafficking is a sensitive subject with employers. Another NGO participant observed that some employers were not receptive to them expanding their remits beyond health to include trafficking, labour conditions or human rights. NGOs might demand that employers improve working or living conditions, or raise awareness among migrant workers about their rights:

“The employer sometimes thinks the NGO is the problem… they don’t need us to be close with the fishermen. Because we will teach them everything. They will understand what is their right. That’s why, when we need something from [employers], if I need information, it’s difficult to go you see. But when I say ‘employer, today we have announcement from MOH, maybe you get vaccine’…If we go and give, the employer allows.” (NGO, 13).

“We just step in about health issues first… [employers] really like that. Not the human rights issue…” (NGO, 7).

NGOs had a better chance of safeguarding access to fishermen via free health services or education, which gave employers benefits in the form of a healthier workforce. To preserve access, NGOs could not be seen to be assisting trafficking cases directly. Instead they referred potential cases to a government unit or another NGO (not from the local area) to conduct the rescue:

“If I go to that area and help a trafficking case, maybe the trafficker will say ‘next time don’t allow this van go to this area’. And now I [provide] HIV training, health education, medicines, then we can get closer with the fishermen. Then we can talk with them “what happened?” If its trafficking or something they can report to us.” (NGO, 13).

This strategy safeguarded NGOs’ access to the area for health promotion and continued monitoring of potential trafficking cases.

### Healthcare and contact with health providers

Most (86.9%) trafficked fishermen could not take time off for sickness or holidays, but two thirds (64.7%) reported ever needing healthcare or being injured (Table 3). Among them, 52.3% said they did not receive care. Most long-haul fishermen (58.2%) were still in pain from their injuries at the time of interview. Among those who received care, one-third (32.0%) said they received some form of care from their manager, 7.3% said they were treated by co-workers and just 6.7% saw a doctor. Following accidents and injuries, lack of first aid knowledge at sea was cited as a problem among health and welfare providers, leading to makeshift self-treatment by fishermen:

“We only saw workers who have been in accidents and are getting complications. For example if a worker got his hand into the boat winch and bled, he would put tobacco paste on it, [causing] swelling or inflammation. Or… if a worker broke his arm he would just put oil on it instead of using a slab to hold it in place. The bone would join themselves back together in 15 days [resulting] in a crooked arm. They do not know how to do it properly… a lot of people get stung by jelly fish and when they don’t do first aid complications will follow. That takes us longer to treat them. That is an important issue.” (HSP, 27).

Self-treatment without proper knowledge could result in long-term harm e.g. wrongly fused bones.

#### Availability

Drop in centres, port outreach and mobile health units ensured availability of primary health services for migrant fishermen. Drop in centres were inviting, including free snacks, books, television; men could access health information, STI testing and Voluntary Counselling and Testing (VCT) for HIV/AIDs. Staff availability was a key theme:

“(This centre) opens at 8 and closes at 5. But we usually we accept people the whole day because the staffs are always here. If you want to drop by then just knock on the door anytime.” (NGO, 15).

One health provider described a pilot Floating Hospital initiative, whereby short-haul fishermen were trained in basic first aid and given medicines to dispense at sea. Participating boats with at least one trained fisherman were given a flag so that other boats could recognise and approach it when men were injured or sick. When boats docked, serious cases were referred to the hospital via mobile health units near ports:

“We have a mobile health unit to do check-ups for crew members. [We diagnose] chronic diseases that are not contagious, diabetes, and high blood pressure. There is also waist measurement to gauge the possibility of being overweight.” (HSP, 27).

Beyond treating injuries, this health provider was concerned about chronic diseases and related risk factors among fishermen. One industry participant described the MOPH doing health checks for infectious diseases among crew on boats returning from international waters:

“… Sometimes they come with diseases… [MOPH] has a space, like the airport… Sometimes the (crew) just walk through, nobody there. [But] if there’s some news about [infectious diseases], now Ebola, if the plane comes from West Africa, then [MOPH] has to come.” (FA, 8).

During a global infectious disease outbreak, where state concerns about disease transmission from mobile groups like fishermen was heightened, checks were more likely to be carried out according to this participant.

#### Health worker attitudes

Staff attitudes and fear of arrest partly governed the decision around which health provider to bring migrant workers. Private clinics where fees could be paid upfront asked fewer questions and were sometimes considered a safer and easier option. This NGO participant suggested that staff at government hospitals were prejudiced against migrant workers who requested fee waivers:

“Sometimes we don’t go to the hospital… We, they [migrant] are scared. We bring them to the sub district hospital… a small treatment centre… If we pay cash they don’t ask too much. If we need assistance from government... Sometimes the hospital (staff) think that the migrant is spending our Thai budget... They don’t like it. Their acting is not human, [like migrants] are not same as their level. They study nurse, the study doctor, but they don’t have heart.” (NGO, 13).

This participant expressed disappointment in health workers who seemed to lack professional ethics in treating migrant workers well, due to inability to pay or perhaps racism. Transporting undocumented migrants in Thailand is illegal, thus getting fishermen to larger, public HSPs entailed personal risk for this participant:

“Sometimes we go to a nearby clinic, pay money and finish… Sometimes we don’t need to bring them far from their home… maybe I will be arrested... Because they are illegal.” (NGO, 13).

Other NGO participants discussed similar fears of arrest and how the law prompted them to be very careful with their work. One participant’s colleague faced criminal charges for transporting an undocumented migrant in their car. Other participants suggested that health workers were welcoming of migrants in healthcare settings, linked to higher volumes of migrants entering Thailand and the ASEAN Economic Community’s (AEC) policy encouraging freer labour movement:

“But I feel like the new doctors that recently graduated are friendly to Burmese because they see more of them and AEC is opening soon. They tell me what they expect from these workers and ask if there is anything they could do to help develop them.” (NGO, 15).

“[Our] hospital is like their ally. [Migrants] can come with or without the money. They are not afraid of this hospital.” (HSP, 27).

In high migration areas where these participants were based, younger doctors and HSPs may be more familiar with and kinder towards migrants seeking care.

#### Paying for treatment

Among trafficked fishermen, 70.9% were cheated of wages and among the few men who received wages these were extremely low (median US$1.44/day) (Table 3), implying that it would be near impossible to pay out-of-pocket for healthcare. Existing long term relationships with HSPs were important when it came to negotiating free treatment, or flexibly paying costs over time, for uninsured migrant workers. When employers refused to pay for treatment, NGOs often had to step in and negotiate with hospitals on fishermen’s behalf:

“We have to check the cost, for example 5000 baht (158 USD). [Fisherman] do not have [that money]. So, that hospital say we only need to pay for the treatment or the medicine... they reduce the cost. So we agree, OK we’ll pay 2000 baht (63 USD). So the hospital say OK… But sometimes it’s very difficult also.” (NGO, 17).

HSPs had discretion to waive fees entirely, or request payment for specific items only. When fees could not be waived entirely, one NGO participant described migrant savings clubs making up the shortfall where fishermen were members. For men in post-trafficking care, hospital invoices would be sent to the Anti-Human Trafficking Fund for payment. Both NGO and government health and welfare providers were concerned about budget constraints when paying treatment costs for uninsured migrants. One HSP noted high awareness among migrants and NGOs about the HSP’s duty to provide free care when needed:

“[Migrants] knew that the government hospitals must give free treatments. We are still doing it now, giving free treatments to illegal foreign workers. In the past 5 years that has cost us 65 million baht (2 million USD). We did not get even a single baht back. 65 million baht, we are in trouble… When we are working with NGO we always request them to help find funding for the government hospital but they always refuse us saying it would be illegal to do so. It is very difficult because it is the government hospital’s responsibility to provide treatment to everyone in need.” (HSP, 27).

This participant highlighted the difficulty of balancing budgets while fulfilling both a legal and moral duty to provide care. Enrolling in the Health Insurance Card Scheme (HICS) was one suggested solution, but fishermen “outside the border” were less likely to enrol than migrants working in other sectors. HICS could only be used for healthcare in Thailand, so long-haul fishermen on boats departing for Indonesia or elsewhere would not be able to use HICS for treatment costs incurred overseas. Other barriers to uptake of HICS included fishermen only being concerned with their health when they had an accident, by which time they would have to pay out-of-pocket. The HSP participant noted that discontinuity of care was common among fishermen because of limited time onshore to have check-ups and get medicines. Some inpatients discharged themselves early to avoid paying for treatment. One NGO participant explained how fishermen were also unlikely to avail of health benefits under the Social Security Scheme (SSS) as they did not enter Thailand via the MOU. Fishermen were entirely dependent on employers’ goodwill to pay out-of-pocket when they were not registered:

“On one boat, there are approximately 40 men, maybe only 2 have documents – pink card [work permit] or passport. Employers don’t normally register them…When they get sick, it depends on how much the employers will take care of them.” (NGO, 25).

NGOs were often contacted by fishermen to help negotiate settlements with employers. One participant discussed numerous challenges in obtaining accident compensation for migrant workers:

“Mostly it’s the social security officers, they try to say that the employers have paid for the treatment, they paid like hundreds of thousand baht… Sometimes they think we are the one who told the employee to be tough. But our duty is to explain to the employees what their losses are, how much they should be compensated. Employees are threatened by employers too. Sometimes we have to help the employees; when they had an accident at work, they cannot work, we have to take care of their rent and food until the case is closed. There are many challenges. Everyone is threatened.” (NGO, 25).

Social security and other government officers also sided with employers during disputes according to other participants, indicating that migrant testimony is not taken seriously by authorities compared to employers’ claims. Employers may threaten migrant employees and NGOs not to pursue compensation claims to avoid pay-outs.

#### Language barriers, interpreters and treatment

Among trafficked fishermen, just 41.1% could speak the language of the destination country (Table 2). Key informants described language barriers as deterring migrant workers from seeking care, or from understanding the benefits of migrant health insurance; one suggested that some health workers’ poor attitudes towards migrants were amplified by frustrations around language:

“Sometimes, the nurse, or official in the hospital sometimes they don’t welcome [migrants]… in my place we don’t have an interpreter in Myanmar or Cambodia language, and then when the nurse shouting them, they don’t want to go. They have problems.” (NGO, 13).

Lack of interpreters in health facilities and the possibility of being reprimanded for language inability discouraged migrants from seeking care. Migrant health volunteer (MHV) interpreters were key in facilitating access to care. An NGO participant who had played a major role initiating a provincial MHV program with the MOPH described high demand for interpreter services. MHVs were not paid, but took pride in their work; they were trained by the NGO and the local public hospital; doctors had translated medical terms to Burmese for the MHV handbook. Being a MHV was a privilege that extended to affording protection from the police, who might otherwise arrest migrant workers:

“Each volunteer will get a Migrant Worker Volunteer shirt… They would let the governor sign their shirts so that the police know they are working with the Health Department and will not arrest them.” (NGO, 15).

The MHV program had extensive support from the provincial health office and governor, indicating that local authorities valued the health of migrants. This support enabled MHVs to operate without having to worry about being arrested. Another participant whose NGO organized their own migrant health interpreters described how employers appreciated this service:

“We have officers and volunteers that can speak Cambodian. We can understand them. When workers are sick, employers send them to the drop in [centre] to let them go to the hospital with volunteers so that they can translate for them. Now the employers saw what we do so they have given us a car to deliver patients. In some cases the workers are sick and they want to go home, we send them all the way to the border with expenses covered by employers.” (NGO, 6).

Employers saw value in interpreter services, engendering further cooperation and a positive relationship with the NGO. Over half of trafficked fishermen (61.7%) were symptomatic of any mental health disorder (Table 4). Key informants also discussed challenges finding interpreters or counsellors speaking native languages in post-trafficking care. One shelter described calling interpreters to translate by phone, or sometimes requesting a resident trafficked person with language skills to interpret. The same shelter usually observed mental health problems among Thai and not migrant residents, for reasons that are unclear but may be related to lack of interpreters to facilitate diagnosis or treatment. One IO provided an additional psychologist and interpreter to support the shelter psychologist with group and individual counselling. Another participant noted that culturally, migrant men dealt with trauma by “getting on with things”, which may be related to treatment in other languages being unavailable:

**Table 4:** Physical and mental health symptoms and concerns post-trafficking among fishermen using post-trafficking services in Cambodia and Thailand (n = 275)

	Long-haul fishermen (*n* = 198)		Short-haul fishermen (*n* = 77)		Whole sample (*n* = 275)	
	Number	Percent	Number	Percent	Number	Percent
Symptom^a^						
Dizzy spells	69	34.9%	14	18.2%	83	30.2%
Headaches	67	33.8%	11	14.3%	78	28.4%
Dental problems	32	16.2%	10	13.0%	42	15.3%
Nausea/indigestion	46	23.2%	10	13.0%	56	20.4%
Diarrhea/gastrointestinal	24	12.1%	8	10.4%	32	11.6%
Back pain	40	20.2%	17	22.1%	57	20.7%
Skin problems	37	18.7%	14	18.2%	51	18.6%
Feeling completely exhausted	63	31.8%	18	23.7%	81	29.5%
Fainting	6	3.0%	2	2.6%	8	2.9%
Significant weight loss	56	28.3%	7	9.1%	63	22.9%
Memory problems	58	29.3%	8	10.4%	66	24.0%
Persistent coughing	33	16.7%	5	6.5%	38	13.8%
Reporting > = 3 areas of pain	62	31.3%	18	23.4%	80	29.1%
Self-assessed health (past month)						
Poor	63	31.8%	11	14.3%	74	26.9%
Fair	100	50.5%	27	35.1%	127	46.2%
Good	34	17.2%	32	41.6%	66	24.0%
Very good	1	0.5%	7	9.1%	8	2.9%
Want to see doctor or nurse for these symptoms#	135/177	76.3%	35/66	53.0%	170/243	70.0%
Post-trafficking mental health^b^						
Symptomatic of depression	122	61.9%	27	35.1%	149	54.4%
Symptomatic of PTSD	94	47.7%	14	18.2%	108	39.4%
Symptomatic of anxiety	106	53.8%	17	22.1%	123	44.9%
Symptomatic of any Mental Health Disorder (MHD)	197	69.5%	77	41.6%	169	61.7%
Self-harm	11	5.6%	3	3.9%	14	5.1%
Suicide attempts	11	5.6%	1	1.3%	12	4.4%
Thoughts of ending your life	18	9.1%	2	2.6%	20	7.3%
Post-trafficking concerns^b^						
Own physical health	73	37.1%	19	24.7%	92	33.6%
Own mental health	36	18.3%	6	7.8%	42	15.3%
Earning money/having job/paying debt	110	55.8%	24	31.2%	134	48.9%
Nowhere to stay short term	17	8.6%	4	5.2%	21	7.7%
Nowhere to stay long term	45	22.8%	12	15.6%	57	20.8%
Money-related problems in family	130	66.0%	40	52.0%	170	62.0%
Health-related problems in family	90	45.7%	38	49.4%	128	46.7%
Afraid of trafficker or associates	11	5.6%	10	13.0%	21	7.7%
Guilt or shame	73	37.1%	19	24.7%	92	33.6%
Documents	15	7.6%	21	27.3%	36	13.1%
Spiritual/religious concerns/ghosts	7	3.6%	7	9.1%	14	5.1%
Other	24	12.2%	23	29.9%	47	17.2%
No concerns	5	2.5%	8	3.5%	13	4.7%
Money concerns (aggregate personal or family)	161	81.7%	47	61.0%	208	75.9%
Post-trafficking concern score (mean)^c^	198	3.2	77	2.9	275	3.1

^a^Proportion endorsing “quite a lot” or “extremely”

^b^One missing for depression, anxiety, each post-trafficking concern, self-harm, suicide attempts among long-haul fishermen/whole sample

^c^Score min = 0, max = 12

“They’re kept in the shelters simply for rehabilitation purposes, which generally fishermen, there’s really not much involved in rehabilitation. For migrants… of course they suffer the trauma, but it’s a very different way of dealing with trauma in this culture, you just get on with things… and the centres are not equipped to provide counselling to people in different languages…They have very basic translators… this is not the kind of advanced stuff, if you’re going to give psychological counselling, you at least need to have decent translators, otherwise how could you do it?” (IO, 2).

For this participant, having professional interpreters, perhaps with medical or specialist knowledge, as opposed to informal interpreters, was considered important to provide appropriate treatment.

### Physical and mental health, post-trafficking concerns

Key informants described seasickness, headaches, muscle pain, fevers and colds as health problems among men at sea, although one HSP noted that it was difficult to know about health problems faced by long-haul fishermen because they did not self-identify as such when seeking care. Among trafficked fishermen, the most commonly reported physical health symptoms were: dizzy spells (30.2%); feeling completely exhausted (29.5%); headaches (28.4%); memory problems (24.0%) (Table 4). If fishermen had escaped trafficking, those who had experienced abuse may experience memory problems:

“Sometimes these people they tend to forget their actual age already because of the continued abuse and exploitation.” (NGO, 7).

Memory problems can complicate repatriation when men forget key information, e.g. names and home addresses. A quarter (26.9%) of trafficked fishermen reported poor self-assessed health and 29.1% reported pain in three or more areas (Table 4). The majority (70.0%) wanted to see a doctor or nurse for their symptoms. Trafficked fishermen had high symptom levels for depression (54.4%), PTSD (39.4%) and anxiety (44.9%); long-haul fishermen had worse mental health than short-haul fishermen; 69.5% of long-haul fishermen were symptomatic of any mental health disorder and 9.1% had suicidal thoughts, compared to 41.6% and 2.6% of short-haul fishermen respectively (Table 4). Money-related concerns (75.9%) and health-related problems in the family (46.7%) were the main post-trafficking concerns among trafficked fishermen (Table 4). Higher proportions of long-haul fishermen were concerned for their physical health (37.1%) and mental health (18.3%), compared to 24.7% and 7.8% respectively among short-haul fishermen. A third (33.6%) were concerned about guilt or shame.

### Factors associated with poor physical health

Among trafficked fishermen, being injured was strongly associated with all physical health symptoms, particularly dizzy spells (UOR 3.39, CI:1.97–5.86) and headaches (UOR 3.13, CI: 1.80–5.43) (Table 5). More severe violence was associated with most physical health symptoms, e.g. dizzy spells (UOR 3.27, CI: 1.65–6.45), poor self-assessed health (UOR 3.41, CI: 1.61–7.20). Being symptomatic of mental health disorders was strongly associated with all physical health symptoms, e.g. pain in three or more areas (UOR 9.00, CI: 4.11–19.68); wide confidence intervals indicate that findings should be interpreted cautiously. Being a long-haul fisherman was associated with most symptoms, e.g. memory problems (UOR 3.57, CI:1.61–7.90). Experiencing more poor living conditions was associated with poor self-assessed health (UOR 2.42, CI: 1.49–3.93); being detained was associated with memory problems (UOR 4.14, CI: 2.30–7.43); being trafficked for more than six months was associated with pain in three or more areas (UOR 3.81, CI: 1.72–8.41). Financial concerns were associated with poor self-assessed health (UOR 2.17, CI:1.06–4.42) and headaches (UOR 2.04, CI: 1.02–4.08). Feeling guilt or shame was associated with poor self-assessed health (UOR 2.08, CI: 1.20–3.60).

**Table 5:** Factors associated with poor physical health of fishermen using post-trafficking services in Cambodia and Thailand (n = 275)

	Dizzy spells (*n* = 83)	Feeling completely exhausted (*n* = 81)	Headaches (*n* = 78)	Memory problems (*n* = 66)	Poor SAH (*n* = 74)	Reporting = > 3 areas of pain (*n* = 80)
	Unadjusted OR	Unadjusted OR	Unadjusted OR	Unadjusted OR	Unadjusted OR	Unadjusted OR
Ever seriously injured^b^	3.39 (1.97–5.86)	3.06 (1.77–5.28)	3.13 (1.80–5.43)	2.26 (1.28–3.99)	2.34 (1.35–4.05)	2.28 (1.33–3.88)
Hours worked/day^c,d^	1.62 (1.01–2.61)	1.09 (0.70–1.71)	1.11 (0.71–1.73)	1.43 (0.83–2.45)	1.78 (1.05–3.00)	0.87 (0.56–1.35)
Occupational Health Risk score^c^	1.25 (0.93–1.69)	1.38 (1.02–1.88)	1.12 (0.82–1.51)	1.14 (0.83–1.57)	1.40 (1.02–1.92)	0.86 (0.64–1.17)
Long-haul fishing^e^	2.40 (1.25–4.60)	1.50 (0.81–2.76)	3.06 (1.51–6.19)	3.57 (1.61–7.90)	2.80 (1.38–5.66)	1.49 (0.81–2.74)
Violence^a^						
Less severe	1.69 (0.69–4.09)	1.66 (0.68–4.03)	1.91 (0.81–4.46)	1.42 (0.54–3.74)	3.13 (1.27–7.72)	1.50 (0.66–3.41)
More severe	3.27 (1.65–6.45)	3.04 (1.53–6.01)	2.36 (1.21–4.63)	2.78 (1.34–5.76)	3.41 (1.61–7.20)	1.80 (0.95–3.41)
Living situation score^c^	1.80 (1.15–2.82)	1.68 (1.07–2.61)	1.40 (0.90–2.18)	1.79 (1.10–2.89)	2.42 (1.49–3.93)	0.96 (0.62–1.47)
Ever detained by immigration authorities^f^	1.47 (0.87–2.48)	2.12 (1.25–3.59)	1.99 (1.17–3.40)	4.14 (2.30–7.43)	1.83 (1.06–3.14)	2.16 (1.27–3.66)
Symptomatic of any MHD^g^	9.67 (4.42–21.15)	3.43 (1.85–6.36)	5.47 (2.72–10.99)	6.33 (2.88–13.92)	6.66 (3.14–14.11)	9.00 (4.11–19.68)
Post-trafficking concerns						
Post-trafficking concern score^c^	1.14 (1.01–1.28)	1.18 (1.05–1.34)	1.20 (1.06–1.36)	1.09 (0.96–1.24)	1.22 (1.08–1.38)	1.07 (0.95–1.20)
Financial concerns^h^	1.21 (0.65–2.24)	1.73 (0.89–3.34)	2.04 (1.02–4.08)	2.06 (0.98–4.32)	2.17 (1.06–4.42)	1.72 (0.89–3.33)
Family health problems^i^	1.08 (0.64–1.82)	1.52 (0.90–2.57)	1.15 (0.68–1.96)	0.57 (0.32–1.01)	1.29 (0.75–2.20)	1.20 (0.71–2.02)
Guilt or shame^j^	1.17 (0.68–2.02)	1.23 (0.71–2.12)	1.50 (0.86–2.59)	0.82 (0.45–1.49)	2.08 (1.20–3.60)	0.86 (0.49–1.50)
Spent > 6 months in trafficking	1.83 (0.94–3.53)	1.38 (0.73–2.61)	2.07 (1.04–4.14)	2.78 (1.25–6.19)	1.89 (0.94–3.79)	3.81 (1.72–8.41)

^a^reference group: no violence

^b^reference group: not injured

^c^rescaled on interquartile range distance

^d^among those who specified hours (*n* = 180)

^e^reference group: short-haul fishing

^f^reference group: not detained by immigration authorities

^g^reference group: not symptomatic of any MHD

^h^reference group: no financial concerns

^i^reference group: no family health problems

^j^reference group: no guilt or shame

## Discussion

Trafficked long-haul fishermen experienced a higher burden of serious injuries and poor health than short-haul fishermen in our sample. Qualitative findings raise questions about adapting Personal Protective Equipment (PPE); how might clothing and PPE be adapted to the weather and Thai context so that fishermen are protected? Besides intense physical exertion, unsanitary, cramped living conditions and poor nutrition exacerbate poor health. Following deaths from beriberi (caused by vitamin B1 deficiencies) linked to such conditions among fishermen [58, 60], MOPH issued guidance on beriberi prevention in the sector is welcomed but it remains to be seen how this information will reach captains and fishermen [61]. We know little about what works to reduce occupational injury and uptake of PPE in low and middle income countries [62]; formative research with migrant fishermen should examine feasibility of different culturally appropriate interventions.

Transshipment in long-haul fishing has undoubtedly contributed to vulnerability and has exacerbated health problems of migrant and trafficked fishermen at sea for extremely long periods. Self-treatment or receiving care from superiors was common in our sample; qualitative findings indicated that such treatment took place without accurate health or first aid knowledge. The Floating Hospital model of having first aid trained fishermen with medicines on boats holds promise, particularly for long-haul fishermen whom cannot reach shore on time following injuries. Encouragingly since September 2016, the Thai government has banned transshipment at-sea permanently, and observers on board are now required by law on all Thai overseas commercial fishing vessels. These observers could be trained in first aid and treatment applications for commonly experienced conditions among long-haul fishermen. July 2016 saw the dispatch of the first fisheries observers to the long-haul fishing fleet, but it is unknown whether they had any health background or training [59].

Making health services migrant friendly is important. Our findings corroborate those in other settings; HSPs are torn between budget constraints and duty to provide care, employing strategies like fee waivers and partnering with NGOs to cover treatment costs [63]. Men tend to delay seeking healthcare when ill [64], indicating that preventive interventions, via port outreach and mobile health units, must continue. Improved registration of migrant fishermen is needed; reforms allowing HICS benefits to be portable would help fishermen to access services wherever they dock in Thailand. Employers of long-haul fishermen should be required to purchase international policies when men apply for work permits; the Ministry of Labour could consider including health insurance on the labour inspection checklist. Our findings show that interpreters encouraged care-seeking and built goodwill with employers. The Migrant Health Volunteer model holds promise, although it has only been implemented in two coastal provinces [65]. In another large-scale NGO program, Migrant Field Officers provide similar support alongside negotiating disputes with police, employers and health workers [66]. In Thailand’s civil service, foreigners are limited to low skilled work and cannot officially be hired as professional interpreters. The government recently announced that migrants could work as “language assistants” but it is too early to tell whether and how this policy affects health or support services for migrant or trafficked fishermen [67]. To date, some migrant interpreters have been employed as cleaners by hospitals who find budget lines to do this. The volunteer MHV scheme itself is hampered by sustainability and budgetary concerns [68]. As per systematic review findings on care provision for migrants, informal interpreters (e.g. friends, family) are considered appropriate by providers when clinical situations are uncomplicated (e.g. coughs, fever) [63], but for mental health or serious conditions trained interpreters are important. Despite interpreters’ valuable role in healthcare settings [69], there appears to be little policy discussion about interpreter services in Thailand. Formative pilot research and economic evaluation of different modalities of interpreting (e.g. including use of information technologies, NGO partnerships) using available conceptual tools [70] may be beneficial to ascertain what could feasibly be implemented in the Thai context.

Stark differences in mental health between long and short-haul fishermen suggest pernicious psychological effects of being trafficked for extended periods at sea. Symptoms of depression and PTSD were more common in our sample compared to adult refugees and conflict afflicted populations where prevalence was measured with similar instruments [71]. With few mental health professionals there are limited referral options; in Cambodia, only severe psychiatric cases among trafficked men were referred to health providers [18]. Men may be “unwilling victims” of trafficking and stigma associated with mental health support may influence care seeking [72, 73]; many trafficked Ukrainian seafarers and fishermen did not seek psychological support because they perceived using services as signs of weakness and debilitation [74]. As our findings show, despite the high burden of mental health symptoms, just 15.3% were concerned for their mental health. Mental health is essential in packages of care for trafficking survivors; research is urgently needed to identify culturally appropriate mental health interventions with men that can be implemented by non-professionals in low-resource settings [42].

Fishermen’s financial concerns, as well as guilt or shame, were associated with poor physical health, which itself may be a somatic symptom of serious psychological distress indicated in our findings. Rescued men often return to deprived origin communities where prospects for work and income are limited, prompting many to re-migrate [18]. Feelings of guilt and shame from failure to fulfil breadwinner expectations are common [75]; Cambodian trafficked fishermen report ridicule from family members for not bringing money home, or some return home to find wives remarried, which may complicate men’s access to social support [18, 76] Fishermen returning with many tattoos have been stigmatized as gangsters or troublemakers, with some reporting discrimination in the job market as a result [76]. In Thailand, promising reforms in 2016 allow trafficked persons to work on one year visas with a quicker application process, with the Anti-Human Trafficking fund covering health insurance and medical examinations needed to apply for work permits [77].

Our study has some limitations. We did not ask fishermen about the duration of their trips at sea. Mental health symptoms may be endorsed differently by nationality. Instruments to measure mental health symptoms were not diagnostic but have been used with traumatized refugees in the same region and among post-trafficking service users in Europe [43, 78, 79]. PTSD symptoms should be interpreted with caution as they may be capturing Acute Stress Disorder [42, 80]. We were only able to conduct bivariable analyses due to small sample sizes for the health outcomes and causal diagrams indicated that multivariable analyses would be biased due to the direction of theorized relationships [81]. While we cannot generalize from qualitative findings, they offer important insights about how service providers reach and assist migrant and potentially trafficked fishermen.

## Conclusion

Improving migrant and potentially trafficked fishermen’s health requires greater investment in OSH interventions and migrant friendly health services. Trafficked fishermen face immense ill-health, particularly trafficked long-haul fishermen, yet resources to restore their wellbeing are not commensurate: most services in the Mekong focus on the needs of women and girls [82, 83]. Now is the time to invest in service provision and research on culturally appropriate care and interventions for migrant and potentially trafficked fishermen.

## Additional file

Additional file 1: Conceptual Framework A. Factors influencing physical health of trafficked fishermen. Conceptual Framework B. Factors influencing healthcare responses for migrant or trafficked fishermen in Thailand. (PDF 606 kb)
