# Supplementary material for: “Because if we talk about health issues first, it is easier to talk about human trafficking”; findings from a mixed methods study on health needs and service provision among migrant and trafficked fishermen in the Mekong
Source: Global Health. 2018 May 9;14:45. doi: 10.1186/s12992-018-0361-x (PMC5941587; doi:10.1186/s12992-018-0361-x)
Supplement: Supplementary file 1 — Conceptual Framework A. Factors influencing physical health of trafficked fishermen. Conceptual Framework B. Factors influencing healthcare responses for migrant or trafficked fishermen in Thailand. (PDF 606 kb) [file 12992_2018_361_MOESM1_ESM.pdf]

Conceptual Framework A. Factors influencing physical health of trafficked fishermen

Pre-trafficking	Trafficking stage	Exit from trafficking	Post-trafficking routes<br>RISK OF RETRAFFICKING	Post-trafficking physical health<br>1 month	 3-12 months
*Socioeconomic characteristics<br>- age<br>- education<br>- income<br><br>*Past physical or mental abuse exposures<br><br>*Health knowledge/practices<br><br>*Masculinity and health seeking behavior<br><br>*Perceived health needs<br><br>*Location of home/worksite, relative to HSPs & transport access<br>- opportunity cost of accessing services<br><br>Risk factor for violence:<br>- language barrier<br><br>*Ability to access healthcare (financial, logistical)<br><br>*Self-treatment<br><br>*Rights to care in home country<br><br>*Social and cultural barriers or facilitators	*Occupational harm/exposures<br>- overexposure to elements<br>- badly maintained or no equipment<br>- long working hours<br>- few or no rest breaks<br>- fatigue/sleep deprivation<br>- repeated bending/lifting heavy objects<br>- unstable platforms<br>- exposure to chemicals<br>- serious injuries<br><br>*Poor living conditions<br>- food/water deprivation<br>- restricted space<br>- poor hygiene<br>- isolation at sea, inability to access care<br><br>*Violence exposures<br>- severity and frequency of physical and psychological abuse by superiors<br>- witness of murder<br>- forced drug taking<br><br>*Duration of trafficking situation<br><br>*Risky behaviours ☒<br>- condom use<br>- alcohol/substance abuse<br><br>*Cash payments (frequency and amount)	*Escape<br>- high risk (jump ship/escape at port whilst docked)<br><br>*Outside intervention<br>- police / immigration raid (with/without NGO)<br>- NGO alone<br><br>*Other<br>- paid off debt and allowed to leave<br>- injured and let go	*Find alternative work<br>- e.g. plantations/agriculture<br><br>*Detention<br>- risk of return to boat owner by police<br>- poor healthcare access<br>- unsanitary conditions<br>- duration of detention<br><br>*NGO/shelter<br>- physical health support<br>- mental health support<br>- culturally appropriate services for men<br>- types of assistance offered (e.g. training, business grant)<br>- duration of shelter stay<br>- ability to work whilst in shelter (restricted mobility / inability to work > risk of re-trafficking)<br>- follow-up assistance after leaving shelter (physical health, counseling, grants)<br><br>*No assistance ☒<br>- identified +unassisted (no assistance offered/available, identified but did not need assistance/declined assistance<br>- unidentified and unassisted	*Perceived health needs<br><br>*Masculinity and health seeking behaviour<br><br>*Ability to access healthcare (financial, logistical)<br><br>*Social support (family, shelter)<br><br>*Anticipated stressors<br>- financial (ability to work + earn, work opportunities, family needs, debt)<br>- social (isolation, experience of shame, fear and stigma)<br>- own physical or mental health<br>- exposure to violence on return<br>- unmet needs (above)<br><br>*Future hopes<br>- social (marriage, kids)<br>- financial (job, income)<br>- cultural (ability to fulfill family and social obligations)<br><br>*Feelings of empowerment<br>↕<br>*Mental health<br>- depression<br>- anxiety<br>- PTSD	*Coping responses ☒<br>- Positive and negative (e.g. substance abuse)<br><br>*Recovery environment characteristics<br>- access to health services/mental health support<br>- work opportunities<br>- social support<br><br>*Anticipated stressors<br><br>*Current perceived needs/future hopes/ feelings of empowerment<br><br>*Mental health ☒<br>- depression<br>- anxiety<br>- PTSD
**HEALTHCARE ACCESS AS CITIZEN**	**POLICY AND POLITICAL INFLUENCES OVER HEALTHCARE ACCESS FOR MIGRANTS**<br>- migrant healthcare access policies/legislation – illegality&health-related "deservingness" - perceptions of public health/security risk				**HEALTHCARE ACCESS (CITIZEN OR MIGRANT)**

☒ Relevant to overall conceptual model but will not be included in analyses (due to nature of STEAM data or not feasible)

↔ Anticipated stressors mediate relationship between trafficking exposures and mental health (examined at one time only)

☒ Relevant to overall conceptual model but will not be included in analyses (due to nature of STEAM data or not feasible)

Source: Pocock NS. Occupational risks, health needs and victim identification of trafficked fishermen in the Greater Mekong Subregion (GMS). PhD thesis [London]: London School of Hygiene and Tropical Medicine; 2017.

Conceptual Framework B. Factors influencing healthcare responses for migrant or trafficked fishermen in Thailand

Healthcare responses as citizen	HEALTHCARE RESPONSES AS MIGRANT				Healthcare responses as citizen or migrant
**Pre-trafficking**	**Trafficking stage**	**Exit from trafficking**	**Post-trafficking routes**<br>**RISK OF RETRAFFICKING**	**Post-trafficking health service access**<br>**1 month**	**3-12 months**
*Perceived healthcare needs<br><br>*Rights to care in home country<br><br>*Education, gender, culture, age, income<br>- health knowledge/practices<br>- health seeking behaviour<br><br>*Social and cultural barriers or facilitators<br><br>*Location of home/worksite, relative to HSPs & transport access<br>- opportunity cost of accessing services<br><br>*Ability to pay for healthcare<br><br>*Self-treatment	*Freedom of movement<br><br>*Fear of arrest/deportation<br><br>*Location of worksite, i.e. physical isolation at sea<br><br>*Physical proximity of public/private HSPs & access to transport<br><br>*Ability to pay for care/transport to HSP<br><br>*Working hours, time off for sickness/HSP visits<br><br>*Legal status & possession of documents (e.g. work permits, MHI card, passport) that enable access to care<br><br>*Language skills/translator availability at HSP<br><br>*Perceived healthcare needs<br><br>*Masculinity & health seeking behaviour<br><br>*Knowledge/information on how to access care<br><br>*Healthcare knowledge of co-workers/ boss/ trafficker (when care received is not from health worker), re. type of care/remedy received<br><br>*Irregular migrant worker health insurance entitlements – knowledge of worker and HSP<br><br>*Self-treatment	Escape, outside intervention (NGO, police) or other:<br><br>*Perceived healthcare needs<br><br>*Masculinity & health seeking behaviour<br><br>*Injuries/illness requiring immediate attention<br><br>*Ability to travel to/knowledge of HSP location<br><br>*Ability to pay for healthcare<br><br>*Legal status & possession of documents<br><br>*Self-treatment	*Perceived healthcare needs<br><br>*Language skills/translator availability at HSP<br><br>*Masculinity & health seeking behaviour<br><br>*Alternative work<br>- location of worksite relative to HSPs & transport access<br>- ability to pay for care/transport<br>- legal status & possession of documents<br><br>*Detention<br>- restricted movement<br>- referral mechanisms for health services<br>- perceived deservingness for care & referrals by detention staff<br><br>*Post-trafficking network healthcare<br>- health worker expertise/capacity<br>- mental health support available<br>- services for men<br>- referral mechanisms for specialized health services<br><br>*Self-treatment	*Perceived healthcare needs<br><br>*Masculinity & health seeking behaviour<br><br>*Social and cultural barriers or facilitators<br><br>*Ability to pay for care/transport to HSP<br><br>*Language skills/translator availability at HSP<br><br>*Self-treatment	*Perceived healthcare needs<br><br>*Masculinity & health seeking behaviour<br><br>*Social and cultural barriers or facilitators<br><br>*Ability to pay for care/transport to HSP<br><br>*Recovery environment characteristics<br>- location of home/worksite, relative to HSPs & transport access<br>- mental health support<br><br>*Language skills/translator availability at HSP<br><br>*Self-treatment
	**POLICY AND POLITICAL INFLUENCES OVER HEALTHCARE REPONSES TOWARDS MIGRANTS**<br>- migrant healthcare access rights/ policies / legislation - illegality and health-related "deservingness" - perceptions of public health /security risk<br>**POLICY AND POLITICAL INFLUENCES OVER OHS/LABOUR PROTECTION**<br>- fishing sector regulation - moral worthiness of male migrant's bodies - neoliberal organization of labour				

Source: Pocock NS. Occupational risks, health needs and victim identification of trafficked fishermen in the Greater Mekong Subregion (GMS). PhD thesis [London]: London School of Hygiene and Tropical Medicine; 2017.
